# Regulatory T Cells: Therapeutic Opportunities in Uveitis

**DOI:** 10.3389/fopht.2022.901144

**Published:** 2022-05-25

**Authors:** Andrew YongJae Lee, William Foulsham

**Affiliations:** Department of Ophthalmology, Weill Cornell Medical College, New York, NY, United States

**Keywords:** regulatory T cells, Tregs, uveitis, inflammatory eye disease, T cell plasticity

## Abstract

Regulatory T cells (Tregs) are critical for the maintenance of immune tolerance and the suppression of excessive inflammation. Many inflammatory autoimmune disorders, including autoimmune uveitis, involve the loss of the suppressive capacities of Tregs. Over the past decade, Tregs’ therapeutic potential in uveitis has garnered increasing attention. Specific subsets of Tregs, including TIGIT+ and PD-1+ Tregs, have emerged as potent immunosuppressors that may be particularly well-suited to cell-based therapeutics. Studies have elucidated the interaction between Treg development and the gut microbiome as well as various intracellular signaling pathways. Numerous cell-based therapies and therapeutic molecules have been proposed and investigated using the murine experimental autoimmune uveitis (EAU) model. However, certain challenges remain to be addressed. Studies involving the use of Tregs in human patients with uveitis are lacking, and there are concerns regarding Tregs’ production and purification for practical use, their plasticity towards inflammatory phenotypes, immunogenicity, and tumorigenicity. Nevertheless, recent research has brought Tregs closer to yielding viable treatment options for uveitis.

## 1 Introduction

Regulatory T cells (Tregs) are a subset of CD4+ T cells that play a critical role in maintaining immune tolerance. Dysfunction and/or depletion of Tregs has been implicated in the pathogenesis of autoimmune disorders including Sjogren syndrome, dry eye disease (DED), Behcet disease (BD), and Vogt-Koyanagi-Harada disease (VKH) (1, 2).

## 2 Mechanism of Action

**Figure 1:**
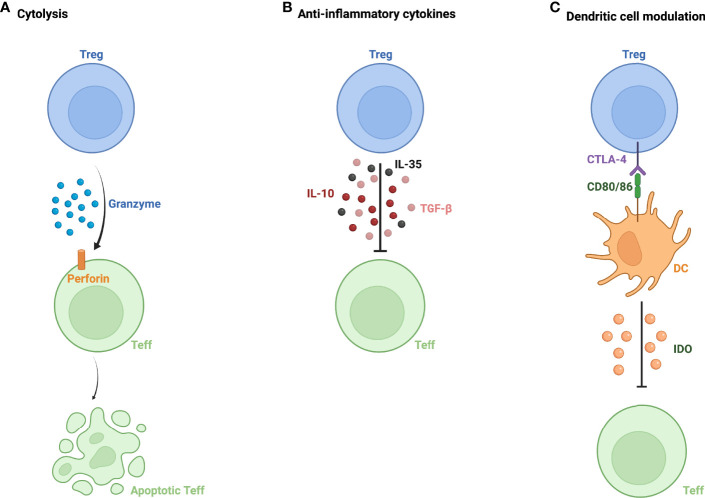
Mechanisms of action of regulatory T cells. **(A)** Granzyme and perforin-mediated apoptosis of effector T cells (Teff) by regulatory T cells (Treg). **(B)** Inhibition of Teff function *via* secretion of anti-inflammatory cytokines- IL-10, IL-35, and TGF-β. **(C)** Modulation of dendritic cells (DC) *via* interaction between CTLA-4 expressed on Treg and DC markers CD80/86, leading to Teff inactivation by DC *via* indoleamine 2,3-dioxygenase (IDO).

Tregs make important contributions to ocular immune privilege, helping to foster a relatively immunoquiescent environment. Tregs utilize multiple mechanisms to exert their immunosuppressive effects, including granzyme-mediated cytolysis, apoptosis of effector T cells (Teff), and the secretion of various anti-inflammatory cytokines including TGF-β, IL-10, and IL-35 (3) (**Figure 1**). Tregs have also been shown to modulate dendritic cells (DCs) (4). The interaction between dendritic cells and Tregs is mediated, in part, by indoleamine (IDO); under inflammatory conditions, IDO activation in antigen presenting cells leads to immune tolerance and inhibition of T cell proliferation (5). Furthermore, Tregs downregulate DC markers CD80/86 and induce apoptosis of DCs *via* IDO (4).

## 3 Classification

Tregs are identified by their surface marker, CD25, as well as their transcription factor forkhead box P3 (FOXP3) (3). However, there are multiple subsets of Tregs, with notable subsets including those that express TIGIT and PD-1. TIGIT+ Tregs promote IL-10 induced proliferation of regulatory dendritic cells, thereby suppressing T cell activation (6). Upregulation of TIGIT expression has been associated with hypomethylation of FOXP3 in Tregs, increasing its expression (7). TIGIT+ Tregs have been implicated in uveitis remission, given the correlation between TIGIT expression and remission in uveitis patients (8). Interestingly, in studies involving the murine experimental autoimmune uveitis (EAU) model, not all Tregs that remain following resolution of uveitis are suppressive (9–12). However, those post-EAU Tregs that maintain their suppressive function have been found to have high levels of TIGIT expression (13). From these data, investigators have suggested that TIGIT might serve as a sensitive marker for remission in uveitis, and that TIGIT might have some utility in identifying ‘functional’ Tregs (8). Yet another subset, PD-1+ Tregs, have similarly demonstrated marked immunosuppressive capacities in the context of autoimmune disorders (14). In uveitis, low levels of PD-1+ Tregs are implicated in developing chronic disease (14). PD-1+ Tregs utilize a melanocortin receptor (MC5r)-dependent pathway, which subsequently activates the adenosine receptor, A2Ar (14). A2Ar stimulation induces Treg proliferation and activation and has been implicated in conferring resistance to EAU relapse (15, 16). Decreased PD-1+ Tregs have been observed in patients with uveitis (14); therefore, targeting A2Ar may represent a potential therapeutic approach, with the goal of bypassing upstream PD-1 and MC5r.

Another area of recent research interest is the enhancers and promoters of FOXP3, called Treg-specific demethylated regions (TSDRs), which influence the level of FoxP3 expression (17). Studies have shown that TSDRs with low methylation levels are crucial for Treg development (18–20). Ten-Eleven-Translocation (Tet) belongs to a family of enzymes that play a role in the demethylation of TSDR (21). Using these concepts, Ito et al. have reported the development of a population of stable, antigen-specific Tregs; the group utilized vitamin C to induce Tet, and subsequent hypomethylation of TSDR within enhancers led to the generation of antigen-specific Tregs with stable FoxP3 expression (17). Given that instability of antigen-specific Tregs has been one of the main barriers to their practical implementation, the ability to maintain stable expression of FoxP3 using this model represents an important step toward clinical use.

## 4 Plasticity

An important consideration in the development of cellular therapies using regulatory T cell concerns their ability to differentiate into pro-inflammatory phenotypes, termed plasticity. Studies have revealed that the transformation of Tregs to T helper 17 cells (Th17) may play a crucial role in the development of various autoimmune disorders (1). This transformation can occur when Tregs are exposed to inflammatory environments, and results in the loss of FOXP3 expression and diminished suppressive ability (1, 22). Moreover, these cells may express pro-inflammatory cytokines, becoming ‘ex-Tregs’ that have been implicated in the pathogenesis of type 1 diabetes, colitis, and arthritis (23). Interestingly, Tregs expressing IL-17 may still have anti-inflammatory effects, depending on the stage of autoimmune disease (24–26). T cells co-expressing FOXP3 and retinoic acid-related orphan receptor (RORγt), a transcription factor for Th17 phenotype, have been shown to produce IL-17, yet these cells continue to suppress T cell proliferation and limit inflammation (27). The mechanism of conversion between phenotypes in Tregs co-expressing CD25 and IL-17 remains unclear at this time; noting that cellular therapies involve transferring cells to pro-inflammatory environments, it is vital to better understand the potential of Tregs to differentiate in these settings.

## 5 Immune Mechanisms in Uveitis

Uveitis is an inflammatory disorder of the eye caused by interactions between ocular autoantigens and T cells (1). T cells that escape negative selection in the thymus migrate to the eye, where they become activated by ocular antigens (28). IFN- γ-producing Th1 and IL-17-producing Th17 cells are the most significant effector T cells driving intra-ocular inflammation (1). In experimental autoimmune uveitis mouse models, Tregs reach their highest frequencies around the peak of disease activity, and remain elevated during resolution, corresponding to increased IL-10 and TGF-β during resolution (29). Unsurprisingly, decreased IL-6 and IL-17 levels are observed during resolution (29). There are conflicting data regarding the correlation between absolute level of Tregs and disease activity in patients with uveitis. While some studies report higher Treg levels in uveitis patients compared to healthy patients, others report lower levels (30–35). More recent studies have proposed measuring Treg/Th17 ratio to assess recovery (29). More specific suppressive Treg markers such as TIGIT and PD-1 may also be valuable in establishing a correlation between Treg level and uveitis resolution.

Current management of uveitis relies heavily on corticosteroids and immunomodulators (36). However, due to adverse systemic and ocular effects seen with prolonged use of these medications, newer therapies with higher efficacy and narrower targets are being sought. Here, we review potential therapies and targets that have been discussed in recently published studies.

## 6 Mouse Models of Uveitis

The role of Tregs in dampening ocular inflammation has been demonstrated in murine models of EAU. Depletion of FoxP3+ Tregs has been shown to result in more severe EAU relative to controls following exposure to IRBP; depletion of Tregs following EAU induction has been shown to inhibit disease resolution, and depletion of Tregs following resolution has been shown to result in relapse (37, 38). These data demonstrate the suppressive function of Tregs at each stage of the disease. Adoptive transfer of FOXP3+ Tregs to mice with established EAU has been shown to reduce inflammation as well as the expression of pro-inflammatory cytokines such as IL-17 and IFN- γ (38, 39). In patients with uveitis, decreased frequencies and impaired function of Tregs has been observed (22).

## 7 Therapeutic Opportunities

### 7.1 Gut Microbiome

The gut microbiome (GM) is known to mediate immunomodulatory effects, and the disturbance of GM has been linked to the development of autoimmune disorders, including uveitis (40–47). Nakamura et al. have documented the difference in composition of GM in patients with uveitis compared to healthy patients (48). The investigators have also demonstrated that antibiotics given orally, but not through intraperitoneal injection, affected susceptibility to EAU induction (48). Vancomycin and Metronidazole, in particular, led to an increase in Treg population in the retina and lymph nodes, associated with decreased EAU severity (48). Other studies have also established reduced incidence of EAU following antibiotic administration in mice (49–52). Kasper et al. investigated the composition of GM in human patients with Behcet Disease (BD), whose GM was characterized by decreased butyrate production (53). Butyrate and other short-chain fatty acids are essential in Treg differentiation and mediate anti-inflammatory effects Novel everolimus-loaded nanocarriers for topical treatment of murine experimental autoimmune uveoretinitis (53). Butyrate has been shown to induce thymic Tregs and suppress pro-inflammatory cytokines (53). Chen et al. studied the effect of sodium butyrate (NaB) administration in EAU (54). NaB resulted in decreased inflammation in EAU, correlated with increased splenic Tregs and a decrease in Th17 (54). NaB-treated mice had increased IL-10 expression and decreased expression of chemokines, IL-17, IFN- γ, TNF-α, and ROR γT (54). The proposed mechanism of NaB involved manipulating the plasticity between Tregs and Th17, redirecting Th17 towards the Treg phenotype by inhibiting IL-6R (54).

### 7.2 Signaling Pathways

Several signaling pathways that affect Treg development have been identified and targeted to study potential therapeutic candidates.

#### 7.2.1 PI3K/AKT

PI3K/AKT pathway promotes secretion of pro-inflammatory cytokines and is essential for Th17 differentiation (55, 56). This pathway is dysregulated in autoimmune disorders, including uveitis (57). Blockage of this pathway using Apremilast, a phosphodiesterase 4 (PDE4) inhibitor, has been used to control inflammation in rheumatoid arthritis, psoriatic arthritis, dermatitis, and other autoimmune disorders (58–63). Chen et al. demonstrated that Apremilast administration results in decreased clinical and histological disease severity in EAU and is associated with increased Treg and decreased Th17 populations (57). Another compound, AS101, a tellurium-based molecule, has been shown to suppress EAU development by inhibiting the activation of AKT (64). Following administration of AS101, increased conversion of naïve T cells to Tregs and decreased Teff population has been observed (64).

#### 7.2.2 STAT

AS101 has also been shown to suppress phosphorylation of STAT3 and STAT4, enzymes of another pathway required for Th17 and Th1 differentiation (64). Wang et al. demonstrated that progranulin (PGRN) induced expansion of antigen-specific and non-specific Tregs *via* STAT5 phosphorylation, reducing EAU severity (65). Correspondingly, decreased PGRN in the peripheral blood of patients with active BD and VKH has been reported (65). Vorinostat, a histone deacetylase inhibitor, has been shown to result in decreased STAT1 and STAT3 expression, associated with increased Treg frequencies (57). Aminooxy-acetic acid (AOA) has also been used to block the STAT/NF-kB pathway, leading to reduced inflammation in EAU (66). As expected, AOA led to upregulation of Tregs and IL-10 and downregulation of Th1/Th17 activity (66).

#### 7.2.3 Notch1

Notch signaling plays an important role in inhibiting Treg differentiation and promoting Teff differentiation (67–70). Exposing Tregs *in vitro* with JAG1 and DLL1, downstream molecules of the Notch1 pathway, has been shown to downregulate FOXP3 expression and modulate the immunosuppressive function of Tregs (71). Furthermore, adoptive transfer of Notch-deficient Tregs in EAU mice has been shown to downregulate pro-inflammatory cytokines in the eye (71). DAPT, a Notch signaling inhibitor, restored Treg/Th17 balance, increased IL-10, and reduced inflammation in EAU (72).

## 8 Cell-Based Therapies

### 8.1 IL-35 and Bregs

IL-35-induced Bregs promote Treg expansion and suppress Th17 and Th1 response in uveitis (73). Adoptive transfer of ex-vivo produced IL-35+ Bregs led to resistance against EAU development and resolution of existing EAU in mice (73). Another study demonstrated that IL-35 containing exosomes secreted from Bregs stimulated IL-10 and IL-35 secreting Tregs, reducing EAU severity (28). Exosomes are particularly beneficial in clinical use because they may be applied topically in the form of an eyedrop and bypass the requirement of generating autologous Bregs for transplant (28).

### 8.2 Col-Tregs

Collagen II is constitutively expressed in the retina and the vitreous (74, 75). Collagen II-specific Tregs (Col-Tregs), activated upon exposure to collagen II, inhibit uveitis in mice (76, 77). Like type 1 Tregs, Col-Tregs display multimodal ability to suppress inflammation (76, 77). Although Col-Tregs have been studied exclusively in mice, they are similar to human Col-Tregs, lack tumorgenicity and plasticity, and minimize systemic immunosuppression, making them promising candidates for therapy (76, 77).

### 8.3 Mesenchymal Stem Cells (MSCs)

Human MSCs are known to exert immunomodulatory effects and generate Tregs (78). MSCs secrete TGF-β and prostaglandin E2 under inflammatory conditions, inducing differentiation of naïve T cells to Tregs (79). MSCs also upregulate their chemokines during inflammation, trapping Th17 cells and shifting them towards the Treg phenotype by taking advantage of their plasticity (80). In addition, MSCs bias macrophages towards the M2 phenotype, which secretes IL-10 and induces Treg expansion (79). Intraperitoneal injection of MSCs in EAU induced antigen-specific Treg development mediated by TGF-β (78). These Tregs remained long after a single injection, conferring protection against EAU relapse (78). However, MSCs are not exclusively anti-inflammatory; they can exert pro-inflammatory effects under certain environmental conditions (79). Therefore, further studies assessing the effect of the microenvironment on MSC phenotype are required.

### 8.4 Human Amniotic Epithelial Cells (hAECs)

hAECs share properties of Col-Tregs and MSCs that make them promising candidates for cell-based therapy in uveitis. hAECs have low immunogenicity (81–83), lack tumorgenicity (84, 85), and can be easily harvested from amniotic membrane (82). Compared to MSCs, hAECs have lower immunogenicity and tumorigenicity and are less prone to apoptosis under inflammatory environments (81, 85). Li et al. revealed that sub-retinal injection of hAECs increased Treg/Th17 ratio by shifting macrophages toward M2 expression, which was associated with a decreased pathological score in EAU (86). Additionally, hAECs were seen to reduce both induction and progression of the disease, depending on when they were administered (day 0 vs. day 6) (86).

### 8.5 Retinoic Acid Receptors (RAR)

RAR is necessary for Treg differentiation and maintenance in inflammatory disease (87–91). RAR contributes to the eye’s immune privilege by inducing differentiation of naïve T cells to Tregs in the aqueous humor (92). Immunization of mice with foreign antigen (to induce EAU) with RAR and low-dose IL-2 induced generation of Tr1 Tregs (93). While Tr1 Tregs do not express FOXP3, they suppress the immune response by secreting IL-10 and expressing immune checkpoints, including CTLA-4 and PD-1 (93). Interestingly, IL-2 has been shown to promote Treg proliferation without affecting Teff (94–96). Therefore, co-administration of RAR and IL-2 can expand Treg *in vivo* and be helpful in therapeutic settings.

### 8.6 Anti-CD4

A recent study by Chen et al. revealed anti-CD4 as a promising tool to generate antigen-specific Tregs *in vivo* (97). *In vivo* generation of stable antigen-specific Tregs has been challenging. This study aimed to address the issue by using anti-CD4 to deplete Teff and then introducing retinal antigen to generate antigen-specific Tregs. Anti-CD4 mediated apoptosis of Teff resulted in increased TGF-β and IL-10 expression, which subsequently led to higher levels of CD25+ FOXP3+ Tregs (97). Introduction of ocular antigens (IRBP and S-antigen, in separate trials) generated Tregs specific to these antigens and suppressed antigen-driven Th17 and Th1 response (97). An overall decrease in inflammation was observed (97). Although additional studies detailing the efficacy and toxicity are required, anti-CD4 represents a step towards the ability to produce Tregs *in vivo*.

## 9 Human Data

No human data demonstrating successful treatment of uveitis with Tregs has been published at the time of this writing (98). However, several therapies that have been used to treat uveitis in humans mediate their effects by induction of Tregs, which are reviewed in this section.

Albayrak et al. investigated the therapeutic effect of IFN-2a on patients with BD (99). The study proposed that IFN-2a leads to recovery of Treg function. 70% of the patients in the study underwent remission of uveitis with a median duration of treatment of 5 months (99). Interestingly, IFN treatment was associated with decreased Treg frequencies, but increased IL-10 and IL-35 secretion by Tregs (99). BD patients had a higher baseline level of Tregs and lower IL-10 than healthy patients. Based on these observations, the authors concluded that Treg dysfunction, rather than scarcity, was contributing to pathogenesis (99). Notably, this is consistent with murine models of ocular inflammation, in which adoptive transfer experiments have shown that far more relevant than Treg frequency is levels of FOXP3 expression, which is directly associated with Treg immunosuppressive function (100).

Given the evidence that the STAT pathway is involved in Treg development and T cell differentiation, JAK inhibitors have been proposed as having potential therapeutic benefit (101). In a case series of four patients with juvenile idiopathic arthritis with uveitis, JAK inhibitors (baricitinib [three cases] and tofacitinib [one case]), successfully suppressed uveitis (101). While tofacitinib has a black box warning for serious infections, malignancies, cardiovascular effects, thrombosis, and all-cause mortality, no such complication was seen in the patient after one year of use. Metformin has also been investigated in BD patients, the use of which has been noted to be correlated with an increase in FOXP3 and TGF-β expression and concurrent decrease in ROR γT, IL-17, and TNF-α (102). Based on reports of IL-2’s ability to expand Tregs without affecting Teff level, Liu et al. administered low-dose IL-2 to BD patients, which led to a reduction in disease severity without adverse effects (103). While all T cell subsets showed expansion under IL-2 stimulation, Tregs underwent the most dramatic increase by a factor of 4 (103).

## 10 Regulatory T Cells in Human Autoimmunity

Strong links between various autoimmune disorders in humans and the deficiency or dysfunction of Tregs lend support to the notion that Tregs are crucial in maintaining immune homeostasis in humans. One notable sequela of Treg dysfunction is X-linked immunodeficiency (IPEX) syndrome, an autoimmune disease that affects multiple organs (104). Mutations in FoxP3, CD25, and CTLA-4, all associated with Treg function, have been implicated in the pathogenesis of autoimmune and inflammatory disorders in humans (105, 106). Importantly, an increase in Th17/Treg ratio has been documented in autoimmune disorders, including multiple sclerosis, psoriasis, rheumatoid arthritis (RA), and inflammatory bowel disease (IBD) (107). Interestingly in both psoriatic and RA patients, Treg level was comparable to that of healthy patients; however, affected patients have Tregs with diminished suppressive capacities, which is thought to be the main contributor to these pathologies (108–110). In psoriatic patients, Tregs were less effective in suppressing IL-17 production and were shown to lose their FOXP3 expression more easily (109, 110).

Given the importance of Th17/Treg imbalance in pathogenesis, several medications are currently undergoing clinical trials to restore their balance to treat MS, RA, SLE, and IBD (107). For instance, in RA patients, monoclonal antibodies against IL-6R and tocilizumab were shown to reduce not only the Th17 phenotype but also increase the Treg population (107, 111, 112). However, there remain controversies regarding the association between Treg level and autoimmunity. There are conflicting data on frequencies of Treg in autoimmune diseases, particularly in SLE and RA. While some studies report an increased level of Tregs in these disease states, others have discovered an unchanged or decreased level of Tregs (113, 114). The discrepant findings expose one of the main challenges to Treg therapy: the ability to identify and target Treg populations reliably. Tregs in SLE have decreased CD25 expression; therefore, using CD25 as a Treg identifier leads to reduced measurements, whereas using FOXP3 leads to increased measurements (115–117). However, a detailed analysis of Treg subpopulations demonstrates a decreased level of ‘effective’ Tregs in both SLE and RA (118). While it is known that multiple subpopulations of Tregs exist in humans, a more reliable and accurate marker to identify different types would be essential in progressing Treg therapy.

As for human therapy utilizing immunomodulatory effects of Tregs, induction of the Treg population with IL-2 has been successful in treating type 1 diabetes, alopecia areata, and hepatitis C virus-induced vasculitis (119). Because Tregs are particularly sensitive to IL-2, IL-2 has been shown to promote Treg proliferation with minimal effects on other T-cell lineages (120). Using an antibody directed against the IL-2 beta chain has been theorized to increase IL-2’s specificity, as Tregs utilize the IL-2 alpha chain for IL-2 binding unlike many other immune cells (120). In addition to IL-2, autologous transfer of *in-vitro* expanded naïve Tregs from human plasma has demonstrated *in-vivo* stability and safety in type 1 diabetes and graft versus host disease patients (121, 122). Further delineation of specific Treg markers and more human data will significantly benefit the potential of Treg therapy in human autoimmunity.

## 11 Discussion, Challenges, and Future Directions

Studies have proposed that ideal characteristics of Tregs for use in therapy include antigen-specificity, maximal purity/avoiding contamination, absence of plasticity toward inflammatory phenotypes, and capacity to migrate to target sites.

Challenges associated with developing antigen-specific Tregs include lack of knowledge of autoantigens that cause autoimmune disorders, and the difficulties associated with producing different sets of Tregs for every autoantigen (49). Given the difficulties, only polyclonal Tregs have been developed for human studies (123). However, polyclonal Tregs are limited in their route of administration and duration of action compared to antigen-specific Tregs. While antigen-specific Tregs have been reported to downregulate ocular inflammation when administered intravenously, the transfer of non-specific Tregs has not been shown to be effective (123). Polyclonal Tregs reduced inflammation only when they were injected *in-situ* (123). In addition, the protection conferred by polyclonal Tregs was transient; recipients were vulnerable to uveitis when Teff were re-introduced at later time points (123). Antigen-specific Tregs, on the other hand, may offer long-term protection. Therefore, there seem to be important advantages of transferring antigen-specific Tregs rather than polyclonal Tregs.

Even when Tregs are generated *in vivo*, they are unstable, limiting their clinical efficacy. Rapamycin, RAR, and IL-2 have been shown to produce stable Tregs, and may become crucial ingredients in the commercial development of Tregs (124). We also discussed that vitamin C-induced hypomethylation of TSDR led to the generation of stable Tregs *in vivo*; in line with this finding, some have suggested epigenetic modification of FOXP3 may be vital to producing a stable population of Tregs (125).

A reliable marker of Tregs must be identified to isolate Tregs from contaminants. Although FOXP3 is a reliable marker, it requires permeabilization, causing cell death (49). Some studies have proposed identifying Tregs with low expression of CD45RA, which corresponds to active Tregs (126, 127). As we mentioned, markers such as TIGIT or PD-1 have been found to represent ‘functional’ suppressive Tregs. As contamination is nearly inevitable even with identifying specific Treg markers, future studies should attempt to uncover the extent to which contamination can be tolerated for clinical use (49).

Finally, we need to better understand Tregs’ plasticity, tumorigenicity, and systemic immunosuppressive capabilities. Tregs have shown a tendency to convert to Th17 phenotype in inflammatory environments with an abundance of IL-6, posing a challenge for treating patients with inflammatory disorders. Some of the mentioned cell-based therapies, especially Col-Tregs (and MSCs and hAECs), which demonstrate low plasticity, tumorigenicity, and systemic immunosuppression, may overcome these challenges.

The role of Tregs in maintaining immune tolerance and as a potential therapeutic tool in uveitis has been actively investigated over the past decade. However, data from human patients are scarce, and barriers to utilizing Tregs in clinical settings remain to be addressed. Future studies addressing these issues and translating EAU studies to human subjects could potentially make cell based Treg therapies a viable treatment option in uveitis.

## Author Contributions

AL and WF: conception of work, drafting and critical revision of work, final approval of the version to be published and agreement to be accountable for all aspects of work.

## Funding

This project was supported in part by a department grant from the Research to Prevent Blindness Foundation.

## Conflict of Interest

The authors declare that the research was conducted in the absence of any commercial or financial relationships that could be construed as a potential conflict of interest.

## Publisher’s Note

All claims expressed in this article are solely those of the authors and do not necessarily represent those of their affiliated organizations, or those of the publisher, the editors and the reviewers. Any product that may be evaluated in this article, or claim that may be made by its manufacturer, is not guaranteed or endorsed by the publisher.
